# The N-terminal domain of Schmallenberg virus envelope protein Gc is highly immunogenic and can provide protection from infection

**DOI:** 10.1038/srep42500

**Published:** 2017-02-13

**Authors:** Kerstin Wernike, Andrea Aebischer, Gleyder Roman-Sosa, Martin Beer

**Affiliations:** 1 Institute of Diagnostic Virology, Friedrich-Loeffler-Institut, 17493 Greifswald - Insel Riems, Germany

## Abstract

Schmallenberg virus (SBV) is transmitted by insect vectors, and therefore vaccination is one of the most important tools of disease control. In our study, novel subunit vaccines on the basis of an amino-terminal domain of SBV Gc of 234 amino acids (“Gc Amino”) first were tested and selected using a lethal small animal challenge model and then the best performing formulations also were tested in cattle. We could show that neither E. coli expressed nor the reduced form of “Gc Amino” protected from SBV infection. In contrast, both, immunization with “Gc Amino”-encoding DNA plasmids and “Gc-amino” expressed in a mammalian system, conferred protection in up to 66% of the animals. Interestingly, the best performance was achieved with a multivalent antigen containing the covalently linked Gc domains of both, SBV and the related Akabane virus. All vaccinated cattle and mice were fully protected against SBV challenge infection. Furthermore, in the absence of antibodies against the viral N-protein, differentiation between vaccinated and field-infected animals allows an SBV marker vaccination concept. Moreover, the presented vaccine design also could be tested for other members of the Simbu serogroup and might allow the inclusion of additional immunogenic domains.

The genus *Orthobunyavirus* which belongs to the family *Bunyaviridae*, the largest and most diverse family of RNA viruses, comprises more than 350 named isolates divided into 18 serogroups^1^, among them the Simbu serogroup. Viruses of this group are distributed worldwide and include e.g. Akabane virus (AKAV), Aino virus, or the recently discovered Schmallenberg virus (SBV) which predominantly infects ruminants and may cause severe fetal malformation when pregnant animals are infected during a critical phase of gestation^2^. Since its emergence in 2011^3^ SBV has caused a large epidemic in European livestock and, starting from the Dutch/German border region, has even spread above the latitude of 65°N. It has infected sympatric wild ruminants grazing alpine meadows at an altitude of more than 2,000 m, was detected as far as Turkey in the southeast and has spread from continental Europe to the British Isles^4^,^5^,^6^,^7^,^8^,^9^. For the prevention of clinical disease, especially premature birth or stillbirth or the induction of fetal malformation, inactivated or live attenuated vaccines have been developed within a short time frame and have been tested successfully^10^,^11^,^12^,^13^. However, vaccines and corresponding test systems which enable differentiation of field-infected from vaccinated animals (DIVA strategy) are still missing. Recent advances in molecular biology and genetics have enabled various new approaches for the development of safe and effective vaccines such as gene deleted, subunit, live-vectored, or DNA-mediated vaccines which could allow marker strategies. However, for all these approaches the identification of at least one antigen or antigenic domain that stimulates a protective immune response is required^14^.

The genome of SBV - as a typical species of the *Orthobunyaviridae* - consists of three negative-sense single-stranded RNA segments designated according to their size as small (S), medium (M) and large (L). The S segment encodes the nucleocapsid protein N and in an alternative overlapping reading frame the non-structural protein NSs; the L segment encodes the viral polymerase, while the M segment encodes a precursor protein which is posttranslationally processed into glycoproteins Gn and Gc and the nonstructural protein NSm^15^. The envelope glycoproteins are targeted by the humoral immune response. Only recently, for SBV a major domain connected to virus neutralization has been identified in the M-segment-encoded Gc protein^16^. This has been discussed as a possible basis of effective vaccines against SBV as well as against related orthobunyaviruses by using the corresponding genomic regions. Providing it is immunogenic, such a domain further represents a promising subunit marker vaccine candidate since antibodies against the N protein would be induced by an SBV field infection and not by vaccination with the Gc domain, and several validated commercial N-based ELISA tests are available which could be used as companion test system.

Therefore, this study aimed to evaluate the immunogenicity of the N-terminal domain of the Gc protein (“Gc amino”) in comparison to full-length SBV-Gc, first in a small animal challenge model system using type I interferon receptor knock-out (IFNAR −/−) mice^11^,^17^, and second in cattle, a major target species of SBV.

However, until now it is not known how the domain must be presented or expressed in order to correctly present the key immunogenic domains. Thus, different approaches were selected: (I) DNA immunization; (II) protein expression in E. coli and a mammalian expression system to examine the influence of glycosylation and other posttranslational modifications; (III) immunization with the non-reduced and the reduced form of the protein to evaluate the influence of disulfide bonds; and (IV) the influence of additionally attached domains and thereby potential use as multivalent vaccines.

## Material and Methods

### Construction of plasmids

As template to perform the PCRs a codon-optimized synthetic gene of the M segment of either SBV or AKAV was used (GeneArt Life Technologies, Regensburg, Germany) unless otherwise indicated. The residue positions are given according to the sequence information of the M protein for SBV (accession number HE649913)^3^ and AKAV (AB100604)^18^, respectively, published in NCBI GenBank. The Phusion HF DNA polymerase used for the PCR and the enzymes applied for cloning were obtained from New England Biolabs (Frankfurt am Main, Germany).

The plasmids encoding for the N-terminal 234 residues of the Gc protein of SBV (SBV-Gc Amino) and for the covalently linked ectodomains of SBV Gn and Gc (SBV-Gn-L-Gc) have been described previously^16^. For bacterial expression of SBV-Gc Amino, a C-terminal hexahistidine tag was added. A PCR product using the plasmid encoding for SBV-Gc Amino as template was generated with the oligonucleotides AATTATT**CCATGG**GTATCAACTGCAAGAACATCCAGAGCACC (restriction sites in bold) and TAATAA**CTCGAG**AATCAGGCTCAGGGTGGTCAGGGTC and subsequently digested with NcoI and XhoI and ligated to the plasmid pGRS-79_Ulm (Roman-Sosa, unpublished) which was also treated with these enzymes. The plasmid was checked by sequencing with the oligonucleotides T7 promoter (TAATACGACTCACTATAGGG) and T7 terminator (GCTAGTTATTGCTCAGCGG).

For DNA immunization, SBV-Gc Amino was used without affinity tag. A PCR product was generated with the oligonucleotides AATTATT**GCTAGC**GCCACCATGGAGACAGACACAC and ATAAAtctagaCTAAATCAGGCTCAGGGTGGTCAG and the plasmid encoding for SBV-Gc Amino as a template. Subsequently the PCR product was restriction digested with NheI/XbaI before being ligated to the pCI Mammalian Expression Vector (Promega, Mannheim, Germany) also processed with these enzymes. The plasmid was checked by sequencing with the primers pCI-fwrd (CCTTTCTCTCCACAGGTGTCCACTCC) and pCI-rev-A (AGCATTTTTT TCACTGCATT CTAGTTG).

The plasmid encoding for the N-terminal 234 residues of the Gc protein of AKAV (construct AKA-Gc Amino) was generated in two cloning steps. Firstly, the gene fragment encoding from Ser 465 to Ile 701 was PCR-amplified with the primers AATTATA**GTCGAC**AGCGCCTGCATCCAGGAAAAAG and **GCT**GATGCTCAGGTCGGGCCGG, the PCR product was restriction digested with SalI and phosphorylated (T4 polynucleotide kinase 3′ phosphatase minus) before being ligated to the plasmid pSignalSeq^16^ treated with SalI and AfeI. Secondly, the plasmid was digested with HindIII and XbaI and the fragment was ligated to the plasmid pEXPR-IBA-103 (IBA, Göttingen, Germany), which was also processed with the same enzymes.

The plasmid encoding for the covalently linked constructs AKAV-Gc Amino and SBV-Gc Amino^16^ (AKA-Gc Amino-SBV-Gc Amino) was likewise generated in two cloning steps. A PCR product was generated with the oligonucleotides ttattaa**CATATG**GATGCTCAGGTCGGGCCGG and AATTATT**TCTAGA**GCCACCATGGAGACAGACACAC using the plasmid encoding for AKA-Gc Amino as template. The DNA fragment was digested with NheI and XbaI and ligated to the plasmid pSignalSeq also processed with these enzymes. Thereafter, the plasmid was digested with XbaI and KpnI and the fragment was ligated to pGRS-19 (Roman-Sosa, unpublished) which also was restriction digested with these enzymes.

The plasmids for the constructs AKA-Gc Amino and AKA-Gc Amino-SBV-Gc Amino were controlled by sequencing with the primers pEXPR-fwrd (GAGAACCCACTGCTTACTGGC) and pEXPR-rev (TAGAAGGCACAGTCGAGG).

### Vaccines

SBV-Gc Amino, AKA-Gc Amino, AKA-Gc Amino-SBV-Gc Amino, or SBV-Gn-L-Gc were ligated to the vector pEXPR-IBA103 and expressed in transiently transfected human embryo kidney cells (HEK-293T, L1018 Collection of Cell lines in Veterinary Medicine (CCLV)) as previously described^16^. Expression and identity of the proteins were confirmed by SDS–PAGE and Western Blot analysis using an anti-Strep tag mAb (StrepMAB-classic, IBA Biotech) (Supplementary Fig. 1).

To generate the vaccine SBV-Gc Amino red the protein SBV-Gc Amino was treated with 10 mM DTT and 0.2% Triton X-100 (v/v) in buffer W (100 mM Tris-HCl pH 8.0, 150 mM NaCl, 1 mM EDTA) for 5 min at 95 °C, and the cysteines were alkylated with 10 mg/ml iodoacetamide for 15 min at 37 °C.

SBV-Gc Amino was additionally expressed in E. coli strain Rossetta in Luria-Bertani (LB) broth supplemented with Kanamycin and glucose (SBV-Gc Amino E. coli). The bacteria were grown at 37 °C until an OD600 of 0.85 was reached and Isopropyl-β-D-thiogalactopyranosid (IPTG) was added to a final concentration of 1 mM. The culture was incubated for another 3 hours at 37 °C and the bacteria were collected. The bacterial pellet was solubilized in a denaturing buffer (4 M GuHCl; 100 mM NaH_2_PO_4_; 100 mM Tris-HCl; 0.5 M NaCl; pH 8.0). The soluble fraction was collected and applied onto a 1 ml Ni-NTA nickel column (Qiagen, Hilden, Germany). The column was washed with denaturing buffer with 20 mM imidazole. The protein was eluted with 5 column volumes of denaturing buffer with 500 mM imidazole. The fractions were analyzed by SDS-PAGE and the fractions containing the protein were pooled and dialyzed against phosphate buffered saline solution (PBS). In order to confirm its identity the dialyzed protein was separated using SDS-PAGE under reducing conditions and visualized by Coomassie Blue staining. The resulting protein bands (see Supplementary Fig. 1) were excised and processed for mass spectrometry analysis. All protein concentrations were determined using Bradford reagent (BioRad, München, Germany), and aliquots were stored at −80 °C until use.

For DNA vaccination, the N-terminal 234 residues of SBV Gc were ligated to the pCI Mammalian Expression Vector (Promega, Mannheim, Germany) and used directly for immunization of mice (SBV-Gc Amino (DNA)).

For immunization of mice with proteins GERBU ADJUVANT MM (GERBU Biotechnik GmbH, Heidelberg, Germany) and for immunization of cattle POLYGEN™ (MVP Technologies, Omaha, USA) were used as adjuvants. All vaccines are listed in Table 1.

### Serology

#### In-house ELISA

Medium binding ELISA plates (Microlon, 96 W; Greiner, Frickenhausen, Germany) were incubated overnight at 4 °C with one of the proteins used for vaccination followed by three washes using PBS-Tween and a one-hour blocking step. Prediluted sera were incubated for 1 hour at room temperature (RT). Subsequently, the plates were washed as described above and the reactivity was shown by adding the corresponding horseradish peroxidase-conjugated anti-species antibody (anti-bovine IgG or anti-mouse IgG; Sigma-Aldrich, Darmstadt, Germany). After an incubation period of 45 minutes at RT the plates were washed again and the substrate was added. Finally, the reaction was stopped and the OD_450_ was measured. Binding of the antigens to the plate was controlled using an anti-Strep-tag mAb (StrepMABclassic; IBA, Göttingen, Germany). A serum sample obtained from cattle infected experimentally with SBV strain BH80/11^19^ was used as positive control in all in-house ELISAs. The results were expressed as the percentage of the sample OD relative to the positive control OD (S/P*100). Every sample was tested in duplicate.

#### Virus neutralization tests

Micro-neutralization tests against SBV or AKAV were performed as described previously^19^. Evaluation was done by assessment of the cytopathic effect after 3 days. All samples were tested in quadruplicate and the antibody titers were calculated as ND_50_ according to Behrens and Kaerber.

### SDS–PAGE and Western blot analysis

BHK cells (L0164 CCLV) were seeded in 6-well-plates and subsequently either infected with SBV isolate BH80/11 or left uninfected as negative control. Cell lysates were prepared 48 hours post infection. The proteins were separated by SDS-PAGE under non-reducing conditions, transferred onto a nitrocellulose membrane and blocked as previously described^20^. The nitrocellulose membranes were incubated with cattle sera diluted 1/100 in PBS-Tween (0.1% Tween20) overnight at 4 °C followed by an incubation with a horseradish peroxidase-conjugated anti-bovine antibody (Dianova, Hamburg, Germany; diluted 1/20 000 in PBS-Tween) for 1 hour at RT. As a loading control, Beta Actin was detected using an anti-Beta Actin monoclonal antibody (1/10 000, Sigma-Aldrich, Darmstadt, Germany) and a peroxidase-conjugated anti-mouse antibody (1/10 000, Dianova, Hamburg, Germany). Proteins were visualized using the Super Signal West Pico Chemiluminescent substrate (Thermo Scientific, Braunschweig, Germany) and analyzed by the Intas ChemoCam System (Intas Science Imaging Instruments GmbH, Göttingen, Germany).

### Animals and experimental design

The experimental protocols were reviewed by the responsible state ethics commission and were approved by the competent authority (State Office for Agriculture, Food Safety and Fisheries of Mecklenburg-Vorpommern, Rostock, Germany, ref. LALLF M-VTSD/7221.3-1.1-004/12). All experiments were performed in accordance with relevant guidelines and regulations.

### Mice

85 adult IFNAR−/− mice on a C57BL/6 genetic background were obtained from the specific pathogen free breeding unit of the FLI; 81 animals were assigned to 9 groups of 9 mice each and immunized or mock-vaccinated twice three weeks apart (groups M01-M09; Table 1), the remaining 4 mice were kept as environmental controls; male and female animals were distributed equally. Three weeks after the second immunization 3 mice of each vaccine group were euthanized; serum samples were collected and analyzed by in-house ELISAs against SBV-Gc Amino, SBV-Gn-LGc, or AKA-Gc Amino-SBV-Gc Amino. All sera were diluted 1/1000. The remaining 6 animals of each group were inoculated subcutaneously (s.c.) with 100 μl of an SBV strain isolated in 2012 from the blood of a viraemic sheep (BH619/12)^21^ that contained 4 log_10_ 50% tissue culture infective doses (TCID50). This SBV strain represents the most suitable challenge material for the used small animal model, since it turned out to be of very high virulence for IFNAR −/− mice.

All mice were weighed daily for 14 days after infection and examined for clinical signs by veterinarians. At days 2 and 7 after infection blood samples were taken from all mice. Animals showing severe symptoms were euthanized immediately; the remaining animals were sacrificed 3 weeks after challenge infection. At autopsy, blood, spleen, liver, intestine and brain samples were taken. Organ samples were weighed and homogenized in 1 ml serum-free MEM. RNA from 20 μl of blood or 100 μl of tissue homogenate was extracted using the King Fisher 96 Flex (Thermo Scientific, Braunschweig, Germany) in combination with the MagAttract Virus Mini M48 Kit (Qiagen, Hilden, Germany) according to the manufacturer’s instructions and analyzed by an S segment based real-time RT-PCR^22^ with an external standard. Subsequently, the copy number per mg of the corresponding organ sample was calculated. Blood samples taken at autopsy were additionally analyzed by in-house ELISAs.

### Cattle

12 cattle of German domestic breeds were assigned to 3 groups of 4 animals each and vaccinated s.c. twice three weeks apart (groups C01-C03; Table 1); 4 additional cattle were kept as unvaccinated controls (C04). Three weeks after the second immunization all animals were inoculated with 2 × 0.5 ml of an SBV field strain that had only been passaged in cattle^23^. The animals were euthanized four weeks after challenge infection. During the entire study, rectal body temperatures were measured daily and the animals were examined for clinical signs by veterinarians. Two animals each of groups C01 and C03 and three cattle of groups C02 and C04 suffered from trichophytia and were treated with TRICHOVAC LTF 130 (IDT Biologika Animal Health, Germany). A total of 10 animals (2× group C01, 3× group C02, 2× group C03, and 3× group C04) received antibiotics (Baxyl LA 200 [Veyx-Pharma GmbH, Germany] and Metapyrin [Serumwerk Bernburg AG, Germany]) because of acute pneumonia.

Sera were taken at weekly intervals throughout the study and analyzed by a microneutralization test against SBV isolate BH80/11, a commercially available N-based ELISA (ID Screen^®^ Schmallenberg virus Competition, ID vet, France), and in-house ELISAs against the proteins used for immunization. Sera from unvaccinated control animals were tested against all proteins used for immunization of the other animal groups, and against AKA-Gc Amino in in-house ELISAs. Sera of group C03 (AKA-Gc Animo-SBV-Gc Amino) taken on the days of vaccination and three weeks after the second immunization were analyzed additionally by a standard microneutralization test against AKAV and sera obtained 4 weeks after challenge infection from cattle of groups C01, C03, and C04 were analyzed by Western blot.

In addition, blood samples were collected daily during the first 10 days after challenge infection and tested by an S segment based real-time RT-PCR^22^. At autopsy, samples were taken from tonsil, spleen, mandibular and mesenteric lymph nodes and analyzed by real-time PCR.

## Results

### Small animal model: Complete protection conferred by the linked Gc domains of SBV and AKAV

**Figure 1:**
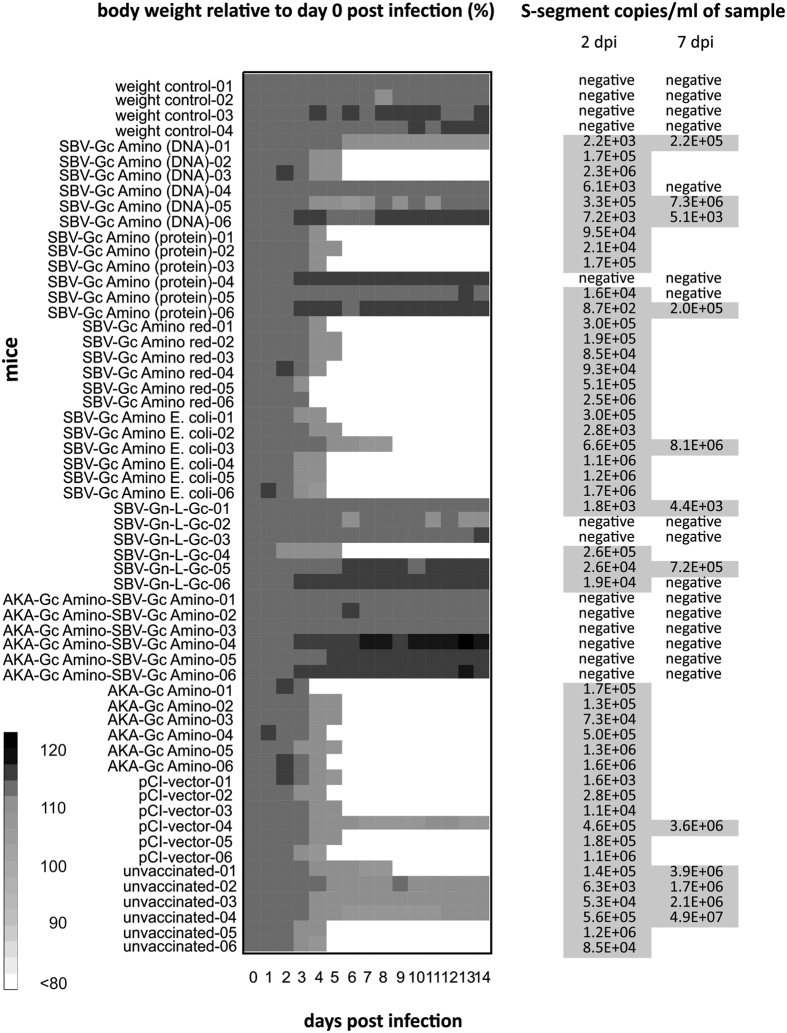
Body weight after challenge infection of mice with SBV and real-time PCR results of blood samples taken 2 and 7 days after infection.

From day two, three or four post challenge infection onwards, all mice of groups M03 (vaccine SBV-Gc Amino red), M04 (SBV-Gc Amino E. coli), M07 (AKA-Gc Amino), M08 (pCI-vector), and M09 (unvaccinated), three animals of group M01 (SBV-Gc Amino (DNA)), three of group M02 (SBV-Gc Amino (protein)), and one mouse of group M05 (SBV-Gn-L-Gc) started to lose weight. Until day 8 after infection, a total of 32 mice (2x M01, 3x M02, 6x M03, 6x M04, 1x M05, 6x M07, 5x M08, 3x M09) were dead or had to be humanely killed (Fig. 1). None of the animals vaccinated with AKA-Gc Amino-SBV-Gc Amino (M06) or the environmental control mice showed any clinical signs. RNAemia was detectable in all animals except the environmental controls, all mice of group M06, two of group M05, and one mouse of group M02 (Fig. 1).

Organ samples taken from deceased mice were highly positive in the SBV-specific real-time PCR (Supplementary Fig. 2). In 13 out of 26 surviving mice, SBV genome was detectable in at least one organ sample at the end of the study. Exceptions were one animal each of groups M01, M02, M05, all six mice of group M06 (Supplementary Fig. 2), and the environmental controls.

Serum samples from mice vaccinated with SBV-Gc Amino either as DNA (M01) or protein (M02-M04) were tested in an in-house ELISA against SBV-Gc Amino; the highest values were obtained with samples from mice which survived the challenge infection (Supplementary Fig. 3). The same holds true for group M05 when tested in an ELISA against SBV-Gn-L-Gc, however, a strong reaction was also detectable for one animal euthanized after the immunizations, but before the challenge infection. An ELISA against AKA-Gc Amino-SBV-Gc Amino was used to test animals of groups M6 and M07; a reaction could be observed in every case. For SBV-infected animals, the highest values were detected in samples of surviving mice (Supplementary Fig. 3).

Sera taken from the control animals (pCI-vector and environmental control mice) were tested against all three antigens; with the exception of the only mouse of group M08 which survived the challenge infection, no reaction to any of the antigens was observed (Supplementary Fig. 3).

### Antibody response of cattle: Novel subunit vaccines are DIVA-compatible

**Figure 2:**
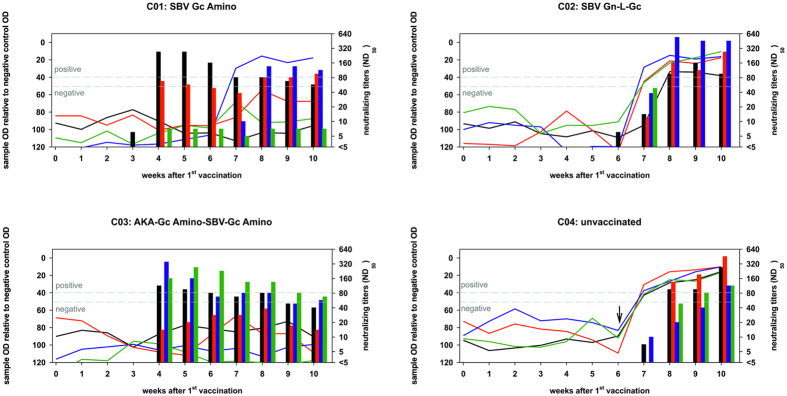
Results of an S-segment based commercial SBV ELISA (depicted by solid lines) and of the micro-neutralization test (shown as bars) of serum samples taken from cattle vaccinated with SBV-Gc Amino, SBV-Gn-L-Gc, AKA-Gc Amino-SBV-Gc Amino, or mock-vaccinated animals. The time point of challenge infection is indicated in the figure panel showing group C04 (unvaccinated) by a black arrow. The results of individual animals in each group are depicted in different colors; the identical colors are used in the corresponding panels of Figs 3 and 5. The cut-off values of the commercial antibody ELISA are marked by a dotted line.

**Figure 3:**
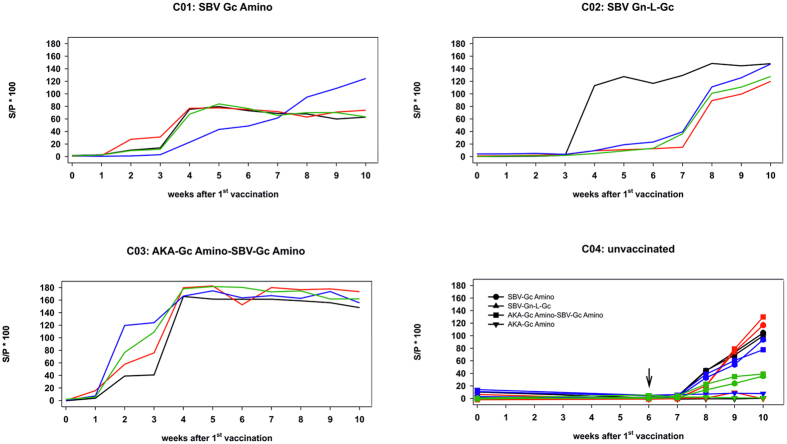
Results of in-house ELISAs of serum samples taken from cattle vaccinated with SBV-Gc Amino, SBV-Gn-L-Gc, AKA-Gc Amino-SBV-Gc Amino, or mock-vaccinated animals. The time point of challenge infection is indicated in the figure panel showing group C04 (unvaccinated) by a black arrow. The results of individual animals in each group are depicted in different colors; the identical colors are used in the corresponding panels of Figs 2 and 5.

In the unvaccinated control cattle no SBV-specific antibodies were detected by any of the applied test systems before challenge infection (Figs 2 and 3); however, neutralizing antibodies were detectable in the control cattle from one or two weeks after SBV infection onwards (Fig. 2); the N-protein-based commercial ELISA scored positive in every case starting from one week after challenge infection (Fig. 2), and the values of the in-house ELISAs against SBV-Gc Amino, SBV-Gn-L-Gc, and AKA-Gc Amino-SBV-Gc Amino increased from week 1 after infection onwards. No SBV-specific reaction was observed against the AKAV domain AKA-Gc Amino (Fig. 3).

In contrast to the unvaccinated control group, SBV-specific neutralizing antibodies were detectable at least two weeks before challenge infection in three out of four cattle immunized with SBV-Gc Amino (group C01; Fig. 2). In these animals, the S/P values of the ELISA against the protein used for immunization increased from one week after the second vaccination (Fig. 3), while the commercial N-protein-based ELISA scored negative until the end of the study (Fig. 2). In the remaining animal of group C01, no SBV-specific neutralizing antibodies could be detected before challenge infection, but from one week after infection, neutralizing as well as N-protein-specific antibodies were present (Figs 2 and 3). The same results were obtained for three out of four animals immunized with SBV Gn-L-Gc (group C02; Figs 2 and 3). In the remaining cattle of group C02, neutralizing anti-SBV antibodies first were detectable on the day of challenge infection (Fig. 2) and the S/P values of the ELISA against SBV Gn-L-Gc increased after the second vaccination (Fig. 3). Positive scores in the N-based ELISA were obtained starting from two weeks after challenge infection onwards (Fig. 2).

**Figure 4:**
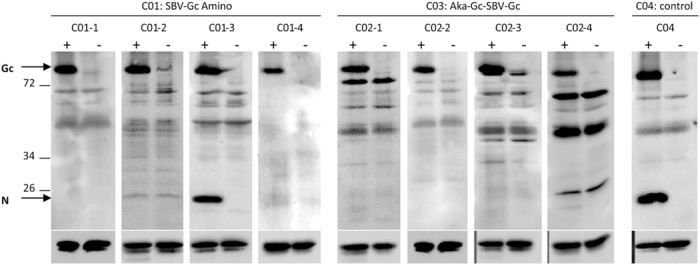
Western Blot analysis. Sera collected at 28 days post challenge from animals of groups C01 and C03 as well as from one control animal from group C04 were analyzed by Western blot using SBV-infected (+) or non-infected cells (−) as antigens. All sera (dilution 1/100) contained Gc-specific antibodies and enabled staining of SBV-Gc (~120 kDa) in infected cell lysates. In contrast, the SBV-N-protein (~25 kDa) was detected only by sera from the infected control animal as well as by one animal from group C01. Staining of Beta Actin was used as a loading control and is shown (cropped) beneath the corresponding serum-staining. Full-length blots are presented in Supplementary Figure 4.

All cattle vaccinated with the concatamer AKA-Gc Amino-SBV-Gc Amino (group C03) developed SBV-specific neutralizing antibodies one week after the second immunization, while they scored consistently negative in the N-protein based commercial ELISA throughout the study (Fig. 2). This could also be demonstrated in Western blot analysis using a whole virus preparation as antigen. As shown in Fig. 4, the N-protein was not detected in serum of any animals from group C03. In the ELISA system specific for the protein used for vaccination, the S/P values increased from two weeks after the first immunization and reached a constantly high plateau phase from one week after the second vaccination (Fig. 3).

All sera of group C03 (vaccine AKA-Gc Amino-SBV-Gc Amino) taken on the day of the first vaccination scored negative in the neutralization test against AKAV. On the day of the second immunization two animals tested negative and two positive (neutralizing titers 1/10 and 1/7) and three weeks after the second vaccination neutralizing antibodies against AKAV were detectable in all four cattle (titers 1/17, 1/34, 1/12, and 1/7).

### Clinical observations and viral RNA detection in cattle: Immunization with the N-terminal domain of the Gc protein protects from viremia

None of the cattle showed any relevant SBV-specific clinical signs during the entire study.

**Figure 5:**
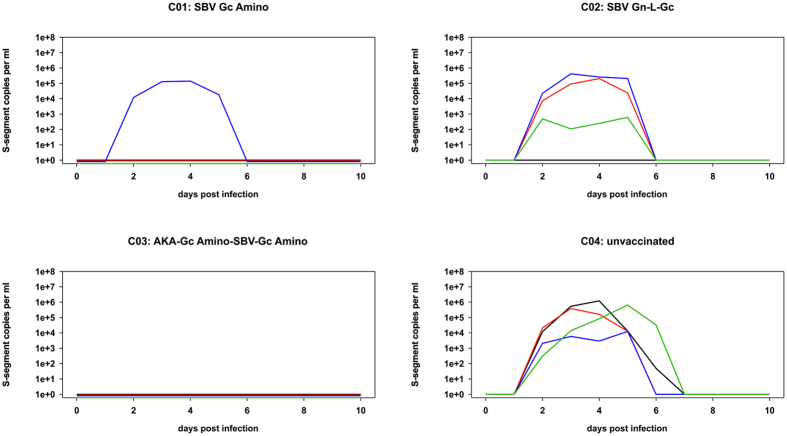
Results of the S-segment based real-time PCR of serum samples taken from cattle vaccinated with SBV-Gc Amino, SBV-Gn-L-Gc, AKA-Gc Amino-SBV-Gc Amino, or mock-vaccinated animals. The results of individual animals in each group are depicted in different colors; the identical colors are used in the corresponding panels of Figs 2 and 3.

Starting from day two post challenge infection, viral RNA was detectable in blood samples of each unvaccinated control animal for four to five consecutive days (Fig. 5). In addition, RNAemia was observed between day two and five in the three animals of group C02 and the one cattle of group C01 in which no neutralizing anti-SBV antibodies could be detected prior to challenge infection (Fig. 5).

In all animals without detectable viraemia also the organ samples tested negative by real-time PCR, whereas in every viraemic cattle SBV genome was found in at least one organ sample (Ct values ranging from 30.4 to 40.5).

## Discussion

Novel members of the family *Bunyaviridae*, the largest and most diverse family of RNA viruses, have been discovered worldwide in the last decade^3^,^24^,^25^,^26^. Moreover, bunyaviruses have spread into previously non-affected regions as frequent detection of these viruses or antibodies against them over the last years has demonstrated. Among these bunyaviruses were also several members of the Simbu serogroup^27^,^28^,^29^ which highlights the need of efficient vaccines against these viruses, especially since they are transmitted by insect vectors and treatment options are not available. Against SBV, which emerged in Central Europe^3^ in late 2011 and subsequently has spread extremely fast throughout the European continent^30^, inactivated and live attenuated vaccines were developed within a short time frame^10^,^11^,^12^,^13^. Even though the relatively inexpensive inactivated vaccines are considered as safe and stable under field conditions, they usually induce weaker or shorter-lived immunity than live vaccines^31^. In turn, live vaccines bear the safety risk of contamination with virulent strains or unwanted pathogens and the potential of reversion to virulence by reversion of attenuating nucleotide mutations^31^,^32^,^33^. In addition, none of the established SBV vaccines enables differentiation of vaccinated from field-infected animals (DIVA).

Apart from inactivated and live attenuated vaccines, live-vectored, DNA-mediated or subunit vaccines, which contain a portion of the infectious agent, are among the most promising antigen delivery systems^31^. However, prior to the selection of the optimal DIVA compatible delivery system at least one antigen that stimulates a robust and protective immune response must be identified and a strategy for its expression and purification must be developed^14^.

The antigenicity of the N-terminal domain of the SBV-Gc-protein has been demonstrated recently^16^, making it a promising basis e.g. for a novel subunit candidate vaccine. However, until now, the immunogenicity of this domain has not been tested *in vivo*. Furthermore, it remains to be elucidated whether the previously described antigenicity depends on correct conformation or specific posttranslational modifications. Generally, the immunogenicity of proteins frequently depends on correct conformation and presentation of the immunogenic protein domain. To facilitate correct folding, these proteins require posttranslational modifications which may only be provided by a eukaryotic expression system^34^. Especially for glycoproteins, the addition of specific carbohydrate components is crucial for folding and antigenicity^35^,^36^. Bacterial expression systems such as E. coli do not have the capacity to glycosylate proteins, since they lack the required cellular organelles^35^,^37^. Nevertheless, E. coli still represents one of the most universal and routinely used protein expression hosts due to its advantages such as a short culture cycle, simple operation, high efficiency and the ability to achieve high expression rates^38^. For these reasons, the E. coli expression system was included in the present study. However, mice vaccinated with proteins expressed in E.coli did not develop a detectable immune response and were not protected against virulent virus challenge. This indicates that correct glycosylation is of vital importance for the antigenicity and immunogenicity of the SBV-Gc-Amino domain, rendering E.coli an unsuitable expression system.

When delivering the antigen as DNA, it is processed *in vivo* using the host cellular machinery including all posttranscriptional modifications resulting in the production of native protein conformations and subsequent induction of both, cellular und antibody immune responses^39^,^40^, which in the present study resulted in a similar performance of the HEK-cell expressed Gc-domain and the DNA-mediated vaccine in immunized mice, indicating correct presentation of the antigen in both delivery systems. Furthermore, as demonstrated by the loss of immunogenicity upon reduction, the maintenance of disulfide bonds within the Gc-Amino domain seems to be crucial for correct presentation of the antigen.

Another important item in terms of correct folding seems to be the insertion of artificial linker sequences between different proteins or domains. The construct SBV-Gn-L-Gc protected only one out of three cattle and two out of six mice from viraemia after SBV infection. This is in contrast to a previously described subunit vaccine based on the Gn and Gc glycoproteins of Rift Valley fever virus, another bunyavirus, which conferred sterile protection of sheep against virulent virus challenge^41^. Therefore, it must be anticipated that in the present study the key immunogens, most likely the aminoterminal domain of Gc, were not completely accessible using the SBV-Gn-L-Gc construct. There might be a negative interaction with the Gn component of this construct, masking the immunodominant regions of Gc.

A superior performance was provided by the bivalent concatameric antigen AKA-Gc Amino-SBV-Gc Amino in both animal species, cattle and IFNAR−/− mice. Since immunization with the Gc domain of AKAV did not show any cross-reactivity with SBV and did not protect from SBV infection in the present study and an inactivated vaccine against AKAV was likewise ineffective against SBV^42^, the sterile protection conferred by the AKA-Gc Amino-SBV-Gc Amino construct is most likely exclusively caused by the immunogenic SBV domain Gc-Amino and its optimized presentation or stabilization by the linked AKAV domain.

Whether the multivalent vaccine construct would also protect from AKAV-infection must be validated in future studies, but it is highly probable since neutralizing anti-AKAV antibodies were detectable in all vaccinated cattle before challenge infection with SBV and every animal with neutralizing anti-SBV antibodies measurable before challenge infection was fully protected against SBV. The development of antibodies which block infection or viraemia and thus provide a correlate of protection^43^ is also a key attribute for vaccines against Simbu serogroup viruses including SBV and AKAV^10^,^44^, thus the bivalent vaccine AKA-Gc Amino-SBV-Gc Amino most likely would protect against both virus infections. Furthermore, the induction of neutralizing anti-AKAV antibodies demonstrated that the domain design could be transferred efficiently from SBV to AKAV, which makes it probable that this also might be possible for other Simbu serogroup viruses, including the important Aino virus and the zoonotic Oropouche virus. Moreover, it might be possible to link a series of Simbu virus domains or even the immunogenic domains of further pathogens in one multiplexed vaccine formulation. However, the positive effect observed with the SBV-AKAV fusion protein needs to be studied further and confirmed with several other examples of orthobunyaviruses. In addition, parameters like the duration of immunity and whether immunization also confers fetal protection should be addressed in future studies. However, since there was a complete absence of viremia upon challenge infection with SBV, it seems highly unlikely that the virus could be transmitted from the dam to the fetus. It has already been shown that e.g. goat fetuses remained virologically negative when their mothers were protected from viremia by immunization with inactivated AKAV vaccines^45^.

The suitability of the IFNAR −/− mouse model for SBV vaccination studies was demonstrated in direct comparison with cattle, showing equal results in both animals species, i.e. partial protection by SBV-Gc-Amino and SBV-Gn-L-Gc expressed in mammalian cells, and complete sterile immunity conferred by AKA-Gc Amino-SBV-Gc Amino in cattle and in mice. This makes the selected mouse model a handy and versatile tool for SBV vaccine research. Especially as vaccination and challenge experiments in ruminants are complicated due to the need of adequate high containment housing, the mouse model facilitates direct comparison of different vaccine delivering systems and/or expression systems.

In conclusion, the double-construct AKA-Gc Amino-SBV-Gc Amino completely prevented viremia in all vaccinated cattle and mice after subsequent challenge infection with virulent SBV, making it an efficient and safe candidate vaccine. Moreover, as shown in the present study, it enables differentiation between vaccinated and field-infected animals using the detection of the SBV-N-protein by commercial antibody ELISAs. The approach of this study additionally demonstrated that novel safe and efficient vaccines can be produced very quickly without the necessity to handle the infectious virus during the production process which is an advantage compared to the currently available inactivated or live attenuated vaccines. The identification of key immunogenic domains and their crucial structural components enables the selection of a suitable expression system as well as a rapid and relatively inexpensive production of potent and safe vaccines.

## Additional Information

**How to cite this article:** Wernike, K. *et al*. The N-terminal domain of Schmallenberg virus envelope protein Gc is highly immunogenic and can provide protection from infection. *Sci. Rep.* **7**, 42500; doi: 10.1038/srep42500 (2017).

**Publisher's note:** Springer Nature remains neutral with regard to jurisdictional claims in published maps and institutional affiliations.

## Supplementary Material

Supplementary Dataset 1

## Figures and Tables

**Table 1:** Vaccines and animal groups.

vaccine	description	dose	animal group
SBV-Gc Amino (DNA)	N terminal 234 residues of SBV Gc in pCI expression vector	100 μg DNA i.m./mouse	M01
SBV-Gc Amino (protein)	N terminal 234 residues of SBV Gc expressed in HEK-293T cells	20 μg protein s.c./mouse; 50 μg protein/cattle	M02, C01
SBV-Gc Amino red	reduced form of SBV-Gc Amino (protein)	20 μg protein s.c./mouse	M03
SBV-Gc Amino E. coli	SBV-Gc Amino expressed in E. coli	20 μg protein s.c./mouse	M04
SBV-Gn-L-Gc	covalently linked ectodomains of SBV Gn and Gc expressed in HEK-293T cells	20 μg protein s.c./mouse; 50 μg protein s.c./cattle	M05, C02
AKA-Gc Amino-SBV-Gc Amino	covalently linked SBV-Gc Amino and AKAV-Gc Amino expressed in HEK-293T cells	20 μg protein s.c./mouse; 50 μg protein s.c./cattle	M06, C03
AKA-Gc Amino	N terminal 234 residues of AKAV Gc expressed in HEK-293T cells	20 μg protein s.c./mouse	M07
pCI-vector	pCI expression vector	100 μg pCI MEGA (DNA) i.m./mouse	M08
unvaccinated	—	100 μl phosphate-buffered saline s.c./mouse	M09, C04
