# Supplementary material for: The N-terminal domain of Schmallenberg virus envelope protein Gc is highly immunogenic and can provide protection from infection
Source: Sci Rep. 2017 Feb 13;7:42500. doi: 10.1038/srep42500 (PMC5304187; doi:10.1038/srep42500)

**The N-terminal domain of Schmallenberg virus envelope protein Gc is highly immunogenic and can provide protection from infection**

Kerstin Wernike, Andrea Aebischer, Gleyder Roman-Sosa, Martin Beer

Supplementary figure 1: Verification of protein identity.

A) Expression and identity of all proteins expressed in HEK293T cells was confirmed by Western blot analysis. 1µg of each protein preparation as well as a negative control (non-transfected cells) and a positive control (non-related Strep-tagged protein) were separated by SDS-PAGE under reducing conditions. The proteins were visualized by Western blot using an anti-Strep-tag mAb.

B: The purified E.coli-expressed SBV-Gc Amino protein was separated by SDS-PAGE under reducing conditions and visualized by Coomassie Blue staining. The bands were extracted from the gel and processed for mass spectrometry analysis in order to confirm the identity of the protein.


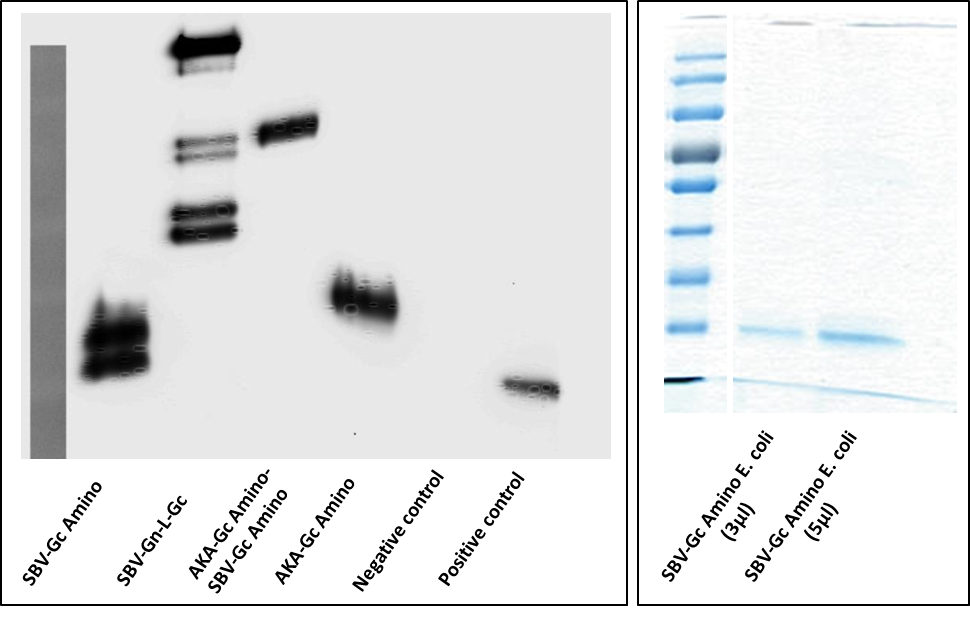


A

B

Supplementary figure 2: Real-time PCR results of tissue and blood samples taken at autopsy, mice that died spontaneously or had to be sacrificed prematurely are marked in red.


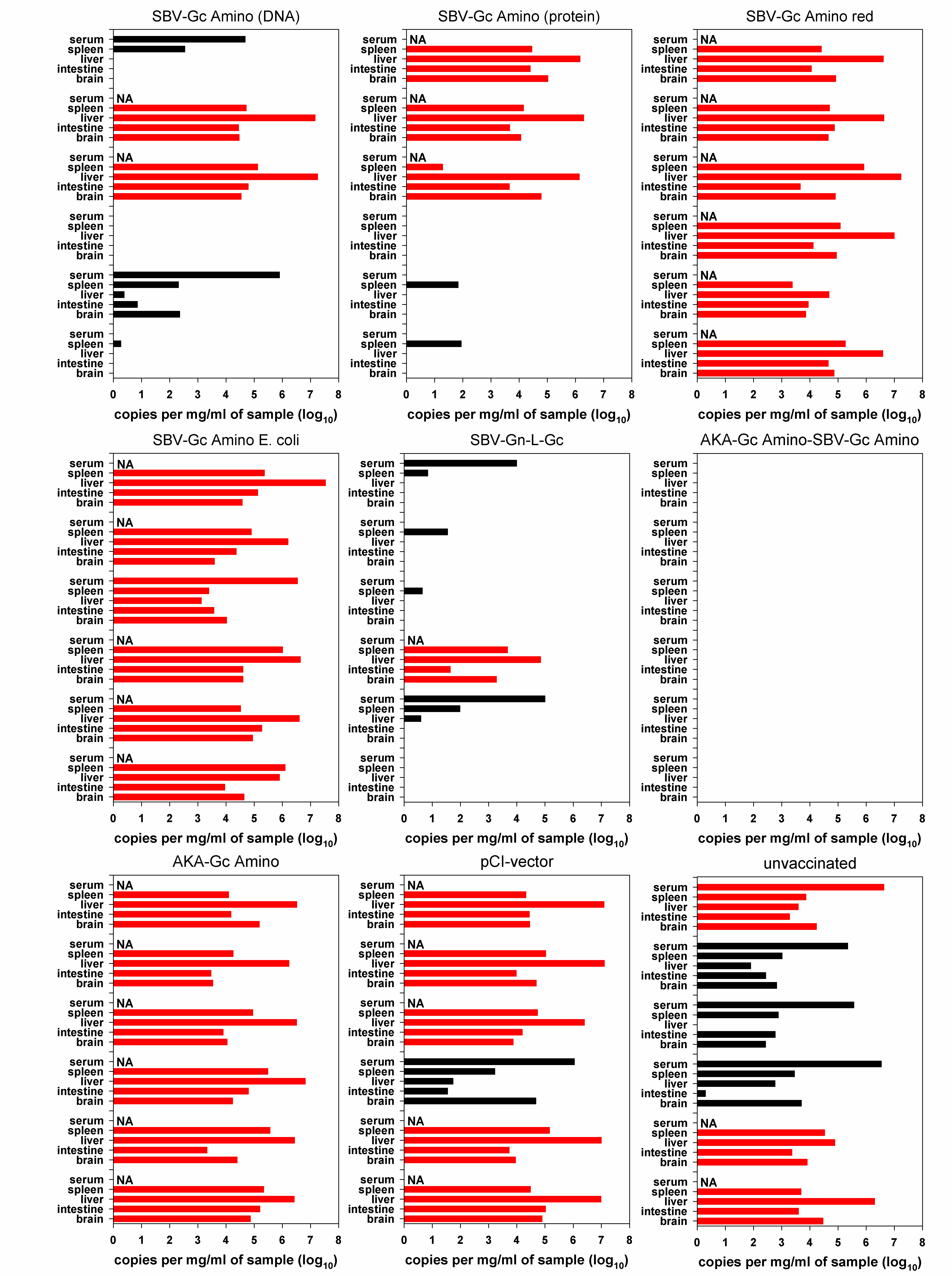


Supplementary figure 3: Antibody response of mice before (“pre”, black dots) and after challenge infection with SBV (“post”), ELISA results of mice which died or had to be euthanized after infection are symbolized by red dots and of surviving mice in red circles. Results of the samples taken from control mice are depicted in green. The number of tested mice per group is shown above of the respective graph.


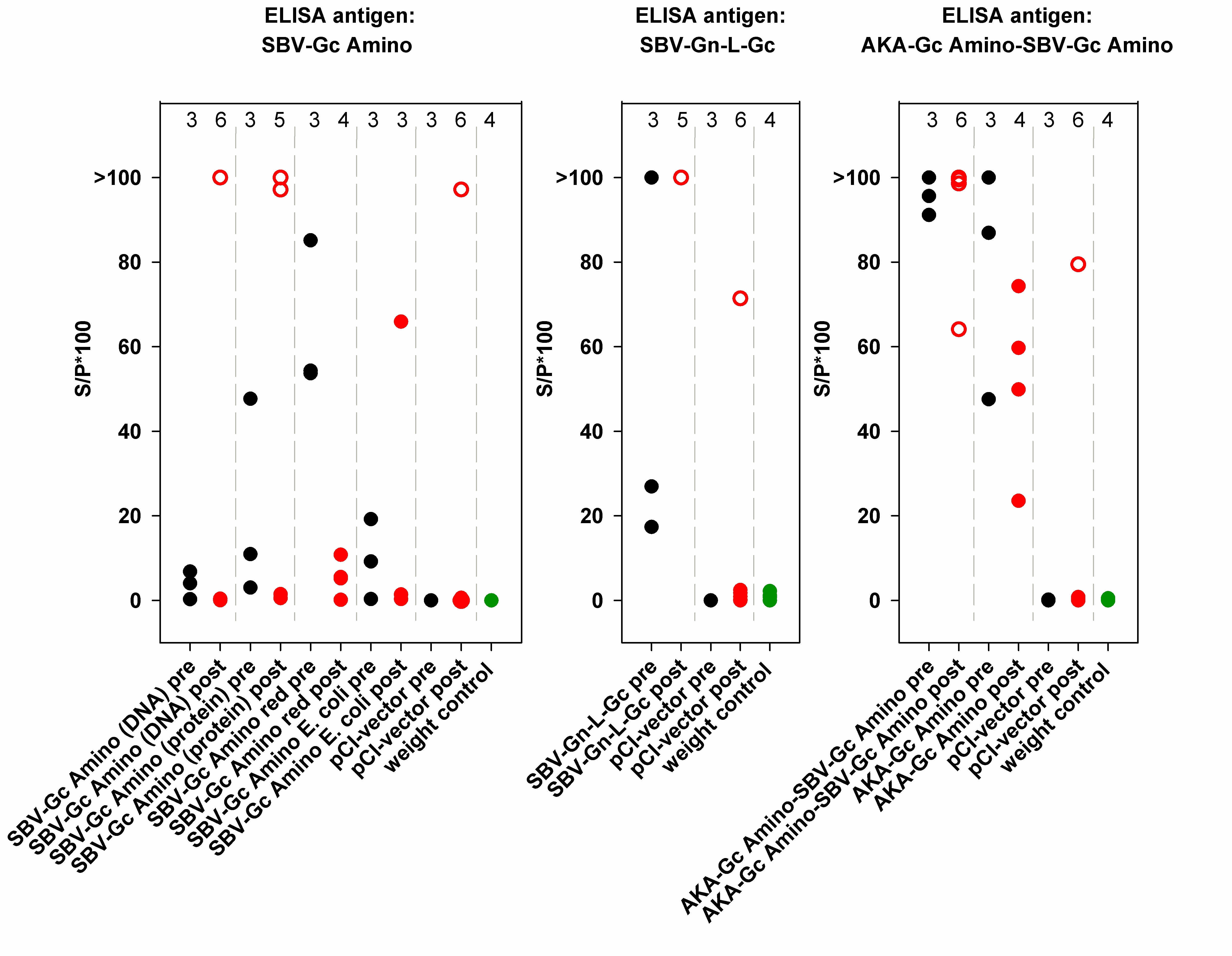


Supplementary figure 4: Full-length blots used for the cropped version of figure 4 are shown.

A: Blots with sera collected at 28 days p.chall. from animals of groups C01 (animals C01-1, -2, -3, and -4; numbering as in figure 4), and C03 (animals C03-1, -2, -3, and -4) as well as from one control animal from group C04. The selected sera were part of different plots with different antibodies and antigens. The selected plots are labeled with the animal number as in figure 4. B: Staining of Beta Actin was used as a loading control in all experiments, and the full-length plots of the relevant bands shown in the cropped figure 4 are provided.

Plots, which were part of the same Western blot experiments, but were irrelevant for this study, are indicated with an asterisk.

A


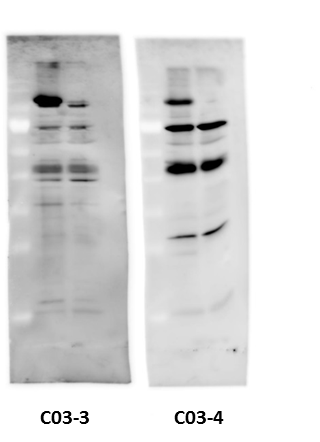

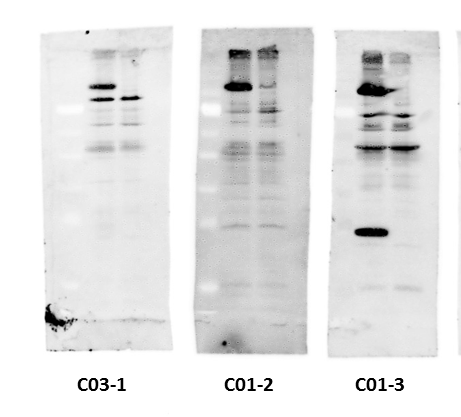

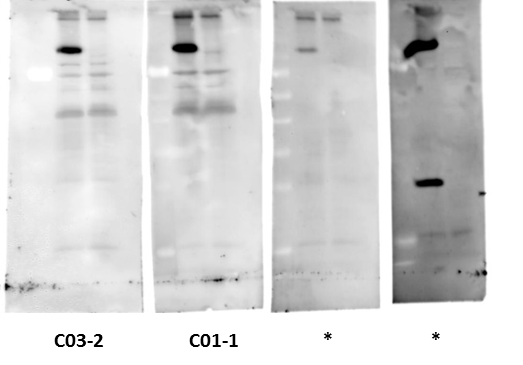

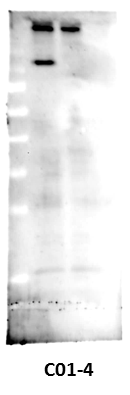

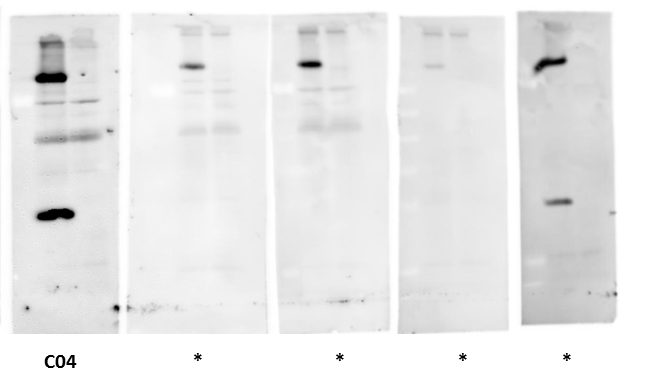


B


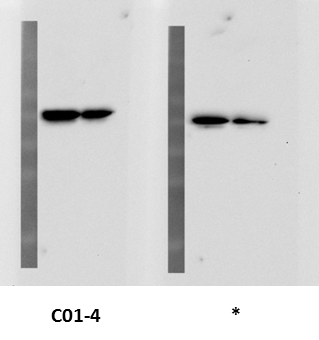

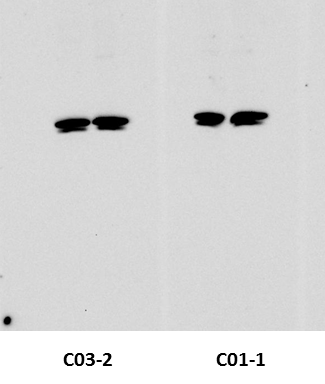


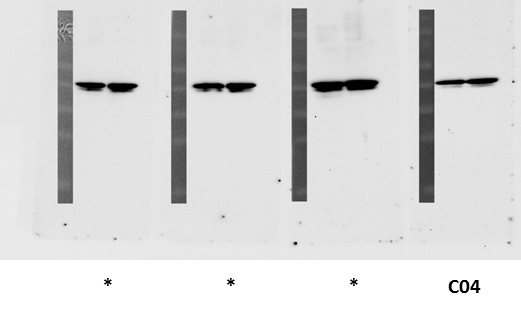

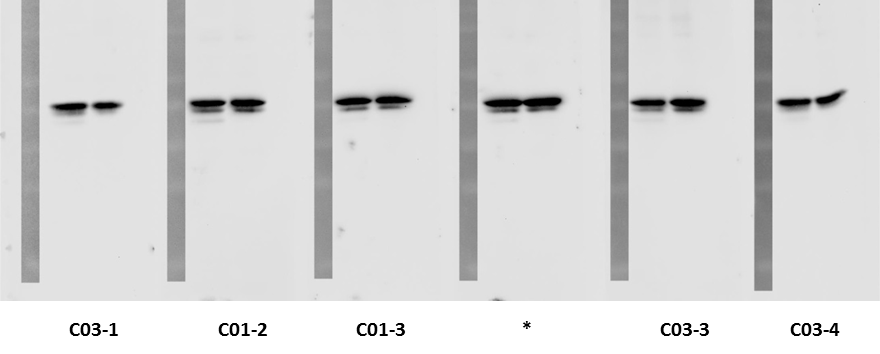

Supplement: Supplementary Dataset 1 [file srep42500-s1.doc]
